# Expression Pattern of Small Nucleolar RNA Host Genes and Long Non-Coding RNA in X-rays-Treated Lymphoblastoid Cells

**DOI:** 10.3390/ijms14059099

**Published:** 2013-04-25

**Authors:** M. Ahmad Chaudhry

**Affiliations:** Department of Medical Laboratory and Radiation Sciences, University of Vermont, Burlington, VT 05405, USA; E-Mail: mchaudhr@uvm.edu; Tel.: +1-802-656-0569; Fax: +1-802-656-2191

**Keywords:** non-coding RNA, TK6 cells, radiation-induced effects

## Abstract

A wide variety of biological effects are induced in cells that are exposed to ionizing radiation. The expression changes of coding mRNA and non-coding micro-RNA have been implicated in irradiated cells. The involvement of other classes of non-coding RNAs (ncRNA), such as small nucleolar RNAs (snoRNAs), long ncRNAs (lncRNAs), and PIWI-interacting RNAs (piRNAs) in cells recovering from radiation-induced damage has not been examined. Thus, we investigated whether these ncRNA were undergoing changes in cells exposed to ionizing radiation. The modulation of ncRNAs expression was determined in human TK6 (p53 positive) and WTK1 (p53 negative) cells. The snoRNA host genes *SNHG1*, *SNHG6*, and *SNHG11* were induced in TK6 cells. In WTK1 cells, *SNHG1* was induced but *SNHG6*, and *SNHG11* were repressed. *SNHG7* was repressed in TK6 cells and was upregulated in WTK1 cells. The lncRNA *MALAT1* and *SOX2OT* were induced in both TK6 and WTK1 cells and *SRA1* was induced in TK6 cells only. Interestingly, the *MIAT* and *PIWIL1* were not expressed in TK6 cells before or after the ionizing radiation treatment. The *MIAT* and *PIWIL1* were upregulated in WTK1 cells. This data provides evidence that altered ncRNA expression is a part of the complex stress response operating in radiation-treated cells and this response depends on functional p53.

## 1. Introduction

The cellular response to ionizing radiation (IR) damage is complex and relies on simultaneous activation of a number of signaling networks. A large number of studies have examined radiation-induced biological effects ranging from DNA damage processing [1], altered gene expression [2,3], cell cycle perturbation [4], bystander effect [5,6], DNA methylation alterations [7], mitochondrial gene expression [8], and microRNA (miRNA) modulation [9].

We and other research groups have published the modulation of miRNA in radiation-treated human cells [10,11]. Later studies from our laboratory examined the impact of radiation dose, dose rate, cellular sensitivity to radiation, and DNA repair capability of the cell on the modulation of miRNA in irradiated human cells [12–15]. In this study, we asked whether other non-coding RNAs (ncRNAs) beside miRNA are modulated in irradiated cells. If so, are there differences or similarities in the ncRNA responses in cells that are sensitive or resistant to radiation-induced killing? We focused on small nucleolar RNAs (snoRNAs) host genes, long ncRNAs (lncRNAs), and PIWI-interacting RNAs (piRNAs) after radiation exposure of human cells. The snoRNAs are a subset of ncRNA with a wide variety of cellular functions, such as chemical modification of RNA, pre-RNA processing and control of alternative splicing [16]. Long non-coding RNAs (lncRNAs) are more than 200 nucleotide in length and lack an open reading frame [17]. LncRNAs are expressed in a disease-, tissue-or developmental stage-specific manner suggesting their specific functions in development and diseases [18]. LncRNAs play an important role in regulating gene expression at various levels, including chromatin modification, transcription and posttranscriptional processing [18,19]. A novel class of small RNAs, called PIWI-interacting RNAs (piRNAs), maintains genome integrity by epigenetically silencing transposons via DNA methylation [20]. piRNAs interact exclusively with the PIWI family of proteins. The piRNAa are aberrantly expressed in a variety of human cancers and in some, its expression correlates with poor clinical prognosis [21].

To elucidate the underlying mechanisms involved in the cellular response to radiation-induced oxidative stress, analyses of the expression of non-protein coding RNA could yield new information. The response of cells to ionizing radiation has been known to result in alterations of gene expression. Our goal was to examine changes in the non-coding RNA expression in cells that are proficient or deficient in *p53*. We investigated if *p53* has an impact on the modulation of non-coding RNA expression in irradiated cells. The p53 protein is a key regulator of gene expression and is essential to maintaining genomic stability [22]. We took advantage of two well characterized lymphoblastoid cell lines TK6 and WTK1 that were derived from the same progenitor cell line WIL2, isolated from a single male donor. TK6 exhibits the wild-type *p53* allele while WTK1 is a *p53* negative mutant [23]. WTK1 cells are more efficient in recombinational repair and have higher resistance to X-irradiation-induced killing than TK6 cells [24]. Alterations in the expression of many nuclear genes [25] and mitochondrial genes [8] have been suggested to contribute to the differences in the response of these cells to IR. Here we report that non-coding RNAs are modulated in these cells after exposure to IR and these changes are correlated with the *p53* status.

## 2. Results and Discussion

### 2.1. Results

We were interested in examining the response of non-coding RNA in TK6 and related WTK1 cell lines after ionizing radiation treatment. We searched the commercially available assays on demand for TaqMan based expression monitoring of ncRNA. A number of ncRNA assays on demand were obtained from Applied Biosystems. The testing of 21 assays in TK6 and WTK1 revealed that 9 of these ncRNA were not expressed in these cell lines. Two of the ncRNA were only expressed in WTK1 cell line. Following these observations we made a short list of 12 ncRNA for further analysis in irradiated TK6 and WTK1 cells. The selection of these ncRNA was purely based on the commercial availability of the reagents for their expression analysis and the observation that these ncRNA were expressed in the cell lines under investigation.

**Figure 1:**
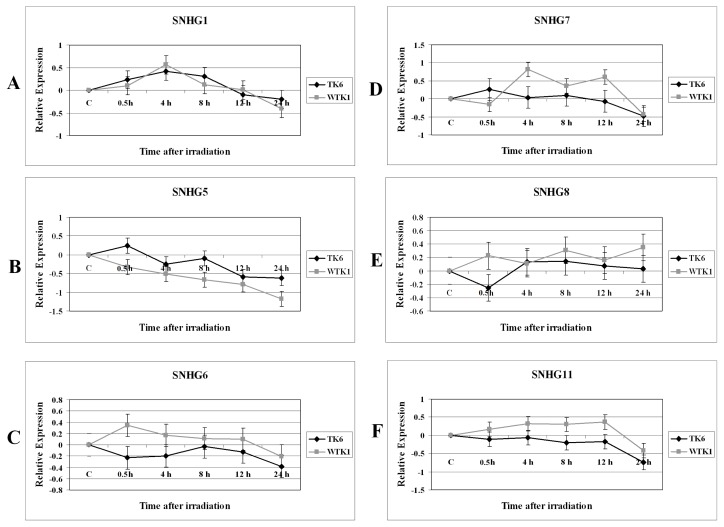
Modulation of various small nucleolar RNA host genes in 2 Gy irradiated TK6 and WTK1 cells. The relative expression, shown as Log_2_ values, was computed at 0.5 h, 4 h, 8 h, 12 h, and 24 h time points. (**A**) *SNHG1*; (**B**) *SNHG5*; (**C**) *SNHG6*; (**D**) *SNHG7*; (**E**) *SNHG8*; (**F**) *SNHG11*. (◆) TK6 cells, (■) WTK1 cells. The error bars indicate the standard error of the mean (SEM) for three independent experiments.

After irradiation with 2 Gy dose of X-rays, the cells were sampled at 0 h, 0.5 h, 4 h, 8 h, 12 h and 24 h time points for investigating the modulation of non-coding RNA. These X-ray treatment conditions have been shown to modulate the expression of several genes and miRNA [15]. Other studies have shown that the expression of p53 changes after X-rays treatment of these cells under similar conditions that we have described [26]. The relative gene expression was determined by the quantitative real-time PCR method. The results obtained with the analysis of various snoRNA host genes *SNHG1*, *SNHG5*, *SNHG6*, *SNHG7*, *SNHG8*, and *SNHG11* expressions are shown in Figure 1.

The *SNHG1*, *SNHG6*, and *SNHG11* snoRNA exhibited similar expression pattern in TK6 and WTK1 cells treated with ionizing radiation. These snoRNAs host genes were initially induced in these cells followed by a decline in their expression level that was observed at late time points (Figure 1A,C,F). In TK6 cells *SNHG1* was induced but *SNHG6*, and *SNHG11* were repressed. *SNHG5* was repressed in irradiated TK6 and WTK1 cells and its expression was further reduced over time after ionizing radiation exposure (Figure 1B). *SNHG7* was unregulated at 4 h and 12 h time points in radiation-treated WTK1 cells (Figure 1D). *SNHG7* was repressed in irradiated TK6 cells (Figure 1D). There were differences in the expression of *SNHG8* in TK6 and WTK1 cells (Figure 1E).

**Figure 2:**
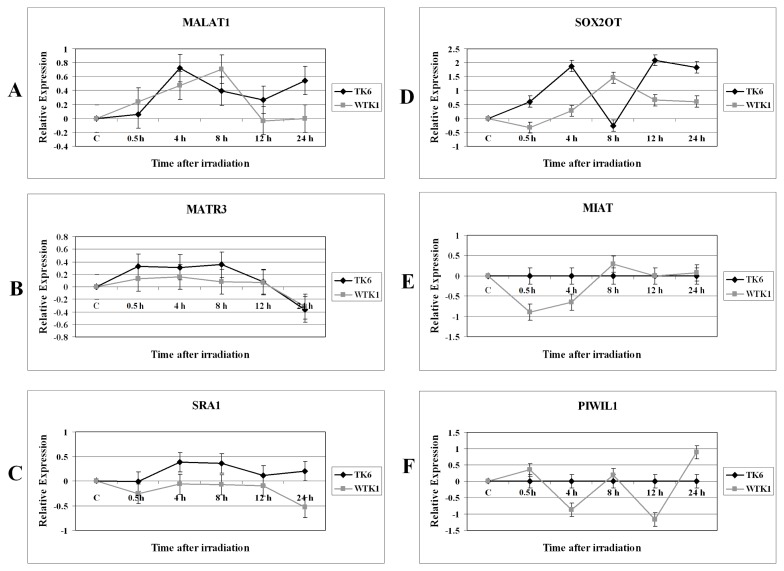
Expression analysis of non-coding RNA genes in 2 Gy irradiated TK6 and WTK1 cells. The data is plotted as Log2 values and indicate relative expression measured at 0.5 h, 4 h, 8 h, 12 h, and 24 h time points after radiation treatment. (**A**) *MALAT1*; (**B**) *MATR3*; (**C**) *SRA1*; (**D**) *SOX2OT*; (**E**) *MIAT*; (**F**) *PIWIL1*. (◆) TK6 cells, (■) WTK1 cells. The error bars indicate the standard error of the mean (SEM) for three independent experiments.

The modulation of *MALAT1*, *MATR3*, *SRA1*, *SOX2OT*, *MIAT*, and *PIWIL1* in irradiated TK6 and WTK1 cells is shown in Figure 2. *MALAT1* was initially induced in both TK6 and WTK1 cells after X-rays treatment (Figure 2A). The expression level of *MALAT1* was declined after 8 h time point in WTK1 cells. The *MATR3* expression was similar in TK6 and WTK1 cells (Figure 2B). The *SRA1* was induced in TK6 cells and after 8 h its expression was repressed (Figure 2C). The expression of *SRA1* remained unchanged in WTK1 cells throughout the time course experiment (Figure 2C) except at the 24 h time point when it was repressed. The difference in the expression of *SRA1* in TK6 cells and WTK1 cells was statistically significant (*p* = 0.0192). The *SOX2OT* was induced in both TK6 and WTK1 cells after treatment with ionizing radiation (Figure 2D). The expression level of *SOX2OT* expression was sharply declined at the 8 h time point in TK6 cells, followed by an increase in its expression level at 12 h time point. In contrast the highest level of *SOX2OT* expression was observed at 8 h time point in WTK1 cells (Figure 2D). The overall expression level of *SOX2OT* was significantly higher in TK6 cells as compared to the WTK1 cells (*p* = 0.018). Finally we monitored the expression pattern of *MIAT* and *PIWIL1* in irradiated TK6 and WTK1 cells. Interestingly the *MIAT* and *PIWIL1* were not expressed in TK6 cells before or after the ionizing radiation treatment (Figure 2E,F). *MIAT* was repressed in WTK1 cells (Figure 2E). *PIWIL1* exhibited two peaks of repression: first at the 4 h time interval, and the second at the 12 h time in irradiated WTK1 cells (Figure 2F). *PIWIL1* was induced at the 24 h time point in WTK1 cells (Figure 2F).

### 2.2. Discussion

We have previously reported the dysregulation of miRNA by radiation [12–15]. Apart from miRNAs, the regulation of expression of other types of ncRNAs in cells treated with ionizing radiation is unknown. Thus, we wondered whether snoRNA host genes, piRNA, and lncRNA were undergoing changes in human cells exposed to IR. We examined selected ncRNA based on the commercial availability of reagents for their analysis. In the absence of a genome-wide expression analysis it is unknown that how many total ncRNAs or miRNA could be expected to display changes in TK6 or WTK1 cells after radiation treatment. The information on the genome-wide mRNA expression is available. A recent publication on microarray based gene expression of TK6 cells exposed to gamma radiation has identified differential expression of 294 genes [27]. The changes in the expression levels of ncRNA observed in the present study are comparable to miRNAs in these cells after irradiation [15].

Alteration of p53 affects cellular responses to DNA damage after treatment with IR. Various cells exhibit a wide range of sensitivities to radiation-induced killing. The p53 negative mutant cell line WTK1 has higher resistance to the toxicity of ionizing radiation than wild type TK6 [26]. Apart from the involvement of p53, the mechanism(s) behind the differential radiosensitivity of TK6 and WTK1 remains unknown. We postulated that a differential ncRNA expression could be responsible for responses to IR in TK6 and WTK1 cells and asked if functional p53 controls the ncRNA expression. We found that the expression patterns of various ncRNAs were different between TK6 and WTK1 cells and these changes could be p53 dependent.

#### 2.2.1. Expression Alterations of Small Nucleolar RNA Host Genes in X-rays-Treated Cells

We first examined the modulation of several small nucleolar RNA host genes (SNHGs) in TK6 and WTK1 cells exposed to 2 Gy of X-rays. SNHGs encode various snoRNA/scaRNA. For example, *SNHG1* encodes U22, U22–U35 boxC/D snoRNAs. *SNHG6* encodes U87 and U88 small Cajal body-specific RNA (scaRNA). Standard nomenclature for snoRNA has not been established. U87 boxC/D snoRNA has also been termed as HBII-276. Apart from SNHG, other ncRNA transcripts, for example MATR3 encodes snoRNA U19 (SNORA74A) box H/ACA. snoRNAs guide the modification of specific nucleotides in ribosomal RNAs (rRNAs) and small nuclear RNAs. A number of tissue-specific snoRNAs have been identified that apparently do not target conventional substrates and are presumed to guide processing of primary transcripts of protein-coding genes [28].

There were similarities and differences in the expression of SNHG in irradiated TK6 and WTK1 cells. The snoRNAs *SNHG1*, *SNHG6*, and *SNHG11* were initially induced in both of these cells followed by a decline in their expression level (Figure 1). In TK6 cells *SNHG1* was induced but *SNHG6*, and *SNHG11* were repressed. *SNHG5* was repressed in irradiated TK6 and WTK1 cells. SNHG5 encodes U50 snoRNA which has been well-characterized as playing an important role in cancer [29]. Future studies of the expression of U50 in irradiated cells will be useful in determining its role in radiation-induced stress pathways. *SNHG7* was upregulated at 4 h and 12 h post radiation treatment of WTK1 cells but was repressed in irradiated TK6 cells. For some SNHG the differences in the expression levels were small. Small differences in the expression levels of certain miRNA have been reported in irradiated cells [10]. Decreased snoRNA expression reduces the snoRNA-guided methylation of the target nucleotides. Impaired rRNA modification, even at a single site, lead to severe morphological defects and embryonic lethality [30]. Because of its central importance, defects in ribosome biogenesis can have detrimental effects on cellular metabolism and vitality. Interestingly, a number of diseases have been associated with defects in ribosome synthesis pathways [31]. We previously reported that exposure of human cells to ionizing radiation results in the modulation of ribosomal genes [32]. We identified 31 ribosomal genes with altered expression in cells irradiated in G1 or G2 cell cycle phases. A small numbers of snoRNAs have been found to play a role in tumorigenesis [29]. snoRNA expression patterns are negatively altered in leukemic cells and prompt cell growth through cell cycle modulation. snoRNA expression was implicated in the G0/G1 to S phase transition mediated by the Rb/p16 pathways [33]. It has been suggested that snoRNAs are targeted by epigenetic inactivation in leukemias. A small number of snoRNAs are overexpressed in lung tumors and act as oncogenes [34].

#### 2.2.2. Expression Changes of Long Non-Coding RNA in Irradiated Cells

Transcriptomic analyses have identified tens of thousands of intergenic, intronic, and *cis*-antisense long non-coding RNAs (lncRNA). The lncRNAs can regulate gene expression at epigenetic, transcription, and post-transcription levels and take part in cell development, immunity, oncogenesis, and clinical disease processes [35]. lncRNAs are dysregulated in a number of human diseases, including several cancers and neurological disorders and show tissue-specific expression [36]. Several lncRNA have increased expression in a number of cancer cells [37]. Genotoxic stress-inducible nuclear lncRNA have been identified [38]. The cigarette smoke exposure and aberrant expression and function of ncRNA has been linked [39]. To our knowledge the modulation in the expression of lncRNA after radiation exposure has not been investigated.

Metastasis-Associated-in-Lung-Adenocarcinoma-Transcript-1 (*MALAT1*) is a lncRNA that is highly expressed in several tumor types and its expression levels are associated with tumor-promoting functions [40]. The function of *MALAT1* is unknown in cells treated with ionizing radiation. *MALAT1* was induced in both TK6 and WTK1 cells after X-rays treatment (Figure 2A). The expression level of *MALAT1* was declined after 8 h time point in WTK1 cells. Other studies have shown that *MALAT1* was down-regulated in bleomycin treated cells [41]. *MALAT1* interacts with pre-mRNA splicing factors and regulates cancer cell migration, synapse formation, cell cycle progression, and responses to serum stimulation. It is proposed that *MALAT1* function becomes apparent under particular conditions [42]. The upregulation of *MALAT1* after radiation exposure suggests its function in DNA damage response.

The steroid receptor RNA activator 1 (*SRA1*) gene encodes both non-coding RNAs and protein-coding isoforms, and they represent alternatively spliced transcript variants. The non-coding *SRA1* is a coactivator for several nuclear receptors (NRs) and is associated with breast cancer [43]. *SRA1* RNA levels affect various biological functions, such as proliferation, apoptosis, steroidogenesis, myogenesis, glucose uptake, cellular signaling, T(3) hormone generation, and invasion/metastasis [44]. The expression of non-coding *SRA1* in irradiated TK6 cells was significantly different from WTK1 cells. The *SRA1* transcript was induced in TK6 cells but its expression was repressed in WTK1 cells (Figure 2C). This observation could be related to the differences in the p53 status among these cells.

The lncRNA *SOX2OT* (*SOX2* overlapping transcript) is embedded within an intron of *SOX2* gene. *SOX2OT* expression has been analyzed in several developmental systems [45]. The involvement of *SOX2OT* in cells recovering from ionizing radiation-induced DNA damage is unknown. The *SOX2OT* was induced in both TK6 and WTK1 cells after treatment with ionizing radiation (Figure 2D). However, there were significant differences in the expression levels of *SOX2OT* among these cells. The expression level of *SOX2OT* was sharply declined at the 8 h time point in TK6 cells, followed by an increase in its expression level at 12 h time point. In contrast the highest level of *SOX2OT* expression was observed at 8 h time point in WTK1 cells (Figure 2D).

The myocardial infarction associated transcript (*MIAT*) does not encode any translational product. Single nucleotide polymorphisms (SNP) at the myocardial infarction (MI) locus on chromosome 22q12.1 are associated with MI. It is suggested that the altered expression of *MIAT* by the SNP may play some role in the pathogenesis of MI [46]. Interestingly the *MIAT* was not expressed in TK6 cells before or after the ionizing radiation treatment (Figure 2). On the other hand *MIAT* was repressed in irradiated WTK1 cells (Figure 2E).

We examined the expression levels of piRNA in TK6 and WTK1 cells subjected to ionizing radiation treatment. piRNAs are 24–30 nucleotides in length, and are synthesized without RNase Dicer participation. piRNAs, form complexes with PIWI proteins, members of the Argonaute family [20]. Interestingly the piRNA, *PIWIL1* (*PIWI-like 1*) was not expressed in TK6 cells before or after the ionizing radiation treatment (Figure 2). *PIWIL1* was repressed at 4 h and 12 h time in irradiated WTK1 cells and was induced at 24 h time point in WTK1 cells. Recent studies have shown that the *PIWIL2* (*PIWI-like 2*) gene is expressed in tumor cells and plays a role in anti-apoptosis as a positive regulator of STAT3 (Signal Transducer and Activator of Transcription 3) signaling [47].

## 3. Experimental Section

### 3.1. Cell Culture and Ionizing Radiation Treatment

The human lymphoblast cell line TK6 was purchased from American Type Tissue Collection (ATCC) (Manassas, VA, USA) and WTK1 cell line was obtained from Dr. Howard Liber, Colorado State University, Fort Collins, CO, USA. These cells were cultured and irradiated as described before [8]. Two Grays dose at 1.7 Gy/min was administered at room temperature. The control sample was treated in the same way, except for irradiation. The treated cells were incubated at 37 °C and harvested at 0.5, 4, 8, 12, and 24 h for isolating RNA. The experiment was repeated in triplicate.

### 3.2. Non-Coding RNA

Assays-on-demand for *SNHG1*, *SNHG5*, *SNHG6*, *SNHG7*, *SNHG8*, *SNHG11*, *MALAT1*, *MATR3*, *SRA1*, *SOX2OT*, *MIAT*, and *PIWIL1* (Table 1) were purchased from Applied Biosystems (Foster City, CA, USA). Standard TaqMan assays have been designed using PrimerExpress software. RNA samples for gene expression analysis were normalized based on the TaqMan Gene Expression Assays for human endogenous hypoxanthine phosphoribosyltransferase (*HPRT*) gene. The sequences of the target regions selected for TaqMan gene expression assays are propriety information that is not disclosed by the manufacturer, Applied Biosystems. Applied Biosystems has assured that the target regions for TaqMan analysis are not overlapped and only host gene specific region is amplified.

### 3.3. cDNA Synthesis, Quantitative Real-Time Polymerase Chain Reaction (QPCR) and Data Analysis

The cDNA was generated from total RNA with random hexamer primers using cDNA synthesis Kit from Applied Biosystems (Foster City, CA, USA) following recommendations of the manufacturer. QPCR was performed on an Applied Biosystems 7900HT Sequence Detection System as described before [8]. The relative expression values of cycle thresholds were calculated by using the comparative delta delta cycle threshold, *ΔΔC_T_* method [48] by normalization to the endogenous control *HPRT* and to the control non-irradiated sample. The sham-irradiated control sample was used as calibrator to calculate the relative expression and Log2 values. The significance of difference in the gene expression was determined by student’s *t*-test.

## 4. Conclusions

The ncRNAs might have a role in radiation-induced cellular effects. Our data suggest that lncRNA expression levels are altered during genotoxic stress and respond differentially in p53 positive and p53 negative cells. The higher resistance to the toxicity of ionizing radiation seen in WTK1 cells could be due to deregulation of ncRNA in these cells along with lack of functional p53. WTK1 cells might have acquired “stem-like” epigenetic and signaling characteristics, including global DNA hypo-methylation [7], and miRNA deregulation. The expression responses of several ncRNA are different in p53 deficient WTK1 cells as compared to TK6 cells indicating dissimilar functions in the IR- induced stress response operating in these cells. The role of p53 in regulating cell cycle and apoptosis is well known but the involvement of p53 in other cellular processes such as metabolism and ribosome biogenesis is not clear. Our data indicates the involvement of ncRNA in IR-induced stress response in a p53 dependent manner. Although the expression patterns of ncRNAs were different between TK6 and WTK1 cells, future studies involving the siRNA based downregulation of p53 in TK6 cells will corroborate p53 impact on ncRNA expression following radiation exposure. Radiation-induced effects could be influenced by alterations of ncRNA expression and their cellular stabilities. It is possible that the function of most ncRNA is to refine gene expression to provide cells more flexibility and the ability to quickly respond to environmental changes.

## Figures and Tables

**Table 1:** Non-coding RNA genes analyzed in irradiated TK6 and WTK1 cells.

Assay ID	Entrez Gene ID	Gene Symbol	Gene Name	Transcribed snoRNA
Hs00411543_m1	23642	SNHG1	Small nucleolar RNA host gene 1	U22, U27, U28, U29, U30, and U31 box C/D
Hs00761246_s1	387066	SNHG5	Small nucleolar RNA host gene 5	U50 and U50B box C/D
Hs00417251_m1	641638	SNHG6	Small nucleolar RNA host gene 6	HBII-276 (U87) box C/D
Hs00288592_m1	84973	SNHG7	Small nucleolar RNA host gene 7	ACA17 and ACA43 box H/ACA
Hs01399325_g1	100093630	SNHG8	Small nucleolar RNA host gene 8	ACA24 box H/ACA
Hs00738242_m1	128439	SNHG11	Small nucleolar RNA host gene 11	ACA39 and ACA60 box H/ACA
Hs00273907_s1	378938	MALAT1	Metastasis associated lung adenocarcinoma transcript 1	-
Hs00251579_m1	9782	MATR3	Matrin 3	U19 box H/ACA
Hs00288796_m1	10011	SRA1	Steroid receptor RNA activator 1	-
Hs00415716_m1	347689	SOX2OT	SOX2 overlapping transcript	-
Hs00402814_m1	440823	MIAT	Myocardial infarction associated transcript	-
Hs01041735_m1	9271	PIWIL1	P-element induced wimpy testis like 1	-

## References

[1] Yang N., Chaudhry M.A., Wallace S.S. (2006). Base excision repair by hNTH1 and hOGG1: A two edged sword in the processing of DNA damage in gamma-irradiated human cells. DNA Repair (Amst).

[2] Chaudhry M.A. (2008). Analysis of gene expression in normal and cancer cells exposed to gamma-radiation. J. Biomed. Biotechnol.

[3] Chaudhry M.A. (2006). Radiation-induced gene expression profile of human cells deficient in 8-hydroxy-2′-deoxyguanine glycosylase. Int. J. Cancer.

[4] Chaudhry M.A. (2007). Base excision repair of ionizing radiation-induced DNA damage in G1 and G2 cell cycle phases. Cancer Cell Int.

[5] Chaudhry M.A. (2006). Bystander effect: Biological endpoints and microarray analysis. Mutat. Res.

[6] Chaudhry M.A., Omaruddin R.A. (2011). Mitochondrial gene expression in directly irradiated and nonirradiated bystander cells. Cancer Biother. Radiopharm.

[7] Chaudhry M.A., Omaruddin R.A. (2012). Differential DNA methylation alterations in radiation-sensitive and -resistant cells. DNA Cell Biol.

[8] Chaudhry M.A., Omaruddin R.A. (2012). Transcriptional changes of mitochondrial genes in irradiated cells proficient or deficient in p53. J. Genet.

[9] Chaudhry M.A., Omaruddin R.A., Kreger B., de Toledo S.M., Azzam E.I. (2012). Micro RNA responses to chronic or acute exposures to low dose ionizing radiation. Mol. Biol. Rep.

[10] Chaudhry M.A. (2009). Real-time PCR analysis of micro-RNA expression in ionizing radiation-treated cells. Cancer Biother. Radiopharm.

[11] Templin T., Paul S., Amundson S.A., Young E.F., Barker C.A., Wolden S.L., Smilenov L.B. (2011). Radiation-induced micro-RNA expression changes in peripheral blood cells of radiotherapy patients. Int. J. Radiat. Oncol. Biol. Phys.

[12] Chaudhry M.A., Omaruddin R.A. (2012). Differential regulation of microRNA expression in irradiated and bystander cells. Mol. Biol. (Mosk).

[13] Lhakhang T.W., Chaudhry M.A. (2012). Interactome of Radiation-Induced microRNA-Predicted Target Genes. Comp. Funct. Genomics.

[14] Chaudhry M.A., Sachdeva H., Omaruddin R.A. (2010). Radiation-induced micro-RNA modulation in glioblastoma cells differing in DNA-repair pathways. DNA Cell Biol.

[15] Chaudhry M.A., Kreger B., Omaruddin R.A. (2010). Transcriptional modulation of micro-RNA in human cells differing in radiation sensitivity. Int. J. Radiat. Biol.

[16] Scott M.S., Ono M. (2011). From snoRNA to miRNA: Dual function regulatory non-coding RNAs. Biochimie.

[17] Chen L.L., Carmichael G.G. (2010). Long noncoding RNAs in mammalian cells: What, where, and why?. WIREs RNA.

[18] Wilusz J.E., Sunwoo H., Spector D.L. (2009). Long noncoding RNAs: Functional surprises from the RNA world. Genes Dev.

[19] Mercer T.R., Dinger M.E., Mattick J.S. (2009). Long non-coding RNAs: Insights into functions. Nat. Rev. Genet.

[20] Siomi M.C., Sato K., Pezic D., Aravin A.A. (2011). PIWI-interacting small RNAs: The vanguard of genome defence. Nat. Rev. Mol. Cell Biol.

[21] Siddiqi S., Matushansky I. (2012). Piwis and piwi-interacting RNAs in the epigenetics of cancer. J. Cell Biochem.

[22] Harvey M., Sands A.T., Weiss R.S., Hegi M.E., Wiseman R.W., Pantazis P., Giovanella B.C., Tainsky M.A., Bradley A., Donehower L.A. (1993). *In vitro* growth characteristics of embryo fibroblasts isolated from p53-deficient mice. Oncogene.

[23] Amundson S.A., Xia F., Wolfson K., Liber H.L. (1993). Different cytotoxic and mutagenic responses induced by X-rays in two human lymphoblastoid cell lines derived from a single donor. Mutat. Res.

[24] Xia F., Amundson S.A., Nickoloff J.A., Liber H.L. (1994). Different capacities for recombination in closely related human lymphoblastoid cell lines with different mutational responses to X-irradiation. Mol. Cell Biol.

[25] Tsai M.H., Chen X., Chandramouli G.V., Chen Y., Yan H., Zhao S., Keng P., Liber H.L., Coleman C.N., Mitchell J.B. (2006). Transcriptional responses to ionizing radiation reveal that p53R2 protects against radiation-induced mutagenesis in human lymphoblastoid cells. Oncogene.

[26] Xia F., Wang X., Wang Y.-H., Tsang N.-M., Yandell D.W., Kelsey K.T., Liber H.L. (1994). Altered p53 Status Correlates with Differences in Sensitivity to Radiation-induced Mutation and Apoptosis in Two Closely Related Lymphoblast Lines. Cancer Res.

[27] Meador J.A., Ghandhi S.A., Amundson S.A. (2011). p53-independent downregulation of histone gene expression in human cell lines by high- and low-let radiation. Radiat. Res.

[28] Bratkovic T., Rogelj B. (2011). Biology and applications of small nucleolar RNAs. Cell Mol. Life Sci.

[29] Dong X.Y., Guo P., Boyd J., Sun X., Li Q., Zhou W., Dong J.T. (2009). Implication of snoRNA U50 in human breast cancer. J. Genet. Genomics.

[30] Higa-Nakamine S., Suzuki T., Uechi T., Chakraborty A., Nakajima Y., Nakamura M., Hirano N., Suzuki T., Kenmochi N. (2012). Loss of ribosomal RNA modification causes developmental defects in zebrafish. Nucleic Acids Res.

[31] Holley C.L., Topkara V.K. (2011). An introduction to small non-coding RNAs: miRNA and snoRNA. Cardiovasc. Drugs Ther.

[32] Chaudhry M.A., Chodosh L.A., McKenna W.G., Muschel R.J. (2003). Gene expression profile of human cells irradiated in G1 and G2 phases of cell cycle. Cancer Lett.

[33] Valleron W., Laprevotte E., Gautier E.F., Quelen C., Demur C., Delabesse E., Agirre X., Prosper F., Kiss T., Brousset P. (2012). Specific small nucleolar RNA expression profiles in acute leukemia. Leukemia.

[34] Mei Y.P., Liao J.P., Shen J., Yu L., Liu B.L., Liu L., Li R.Y., Ji L., Dorsey S.G., Jiang Z.R. (2012). Small nucleolar RNA 42 acts as an oncogene in lung tumorigenesis. Oncogene.

[35] Huang Y., Liu N., Wang J.P., Wang Y.Q., Yu X.L., Wang Z.B., Cheng X.C., Zou Q. (2012). Regulatory long non-coding RNA and its functions. J. Physiol. Biochem.

[36] Niland C.N., Merry C.R., Khalil A.M. (2012). Emerging Roles for Long Non-Coding RNAs in Cancer and Neurological Disorders. Front Genet.

[37] Silva J.M., Perez D.S., Pritchett J.R., Halling M.L., Tang H., Smith D.I. (2010). Identification of long stress-induced non-coding transcripts that have altered expression in cancer. Genomics.

[38] Mizutani R., Wakamatsu A., Tanaka N., Yoshida H., Tochigi N., Suzuki Y., Oonishi T., Tani H., Tano K., Ijiri K. (2012). Identification and characterization of novel genotoxic stress-inducible nuclear long noncoding RNAs in mammalian cells. PLoS One.

[39] Maccani M.A., Knopik V.S. (2012). Cigarette smoke exposure-associated alterations to non-coding RNA. Front Genet.

[40] Schmidt L.H., Spieker T., Koschmieder S., Humberg J., Jungen D., Bulk E., Hascher A., Wittmer D., Marra A., Hillejan L. (2011). The long noncoding MALAT-1 RNA indicates a poor prognosis in non-small cell lung cancer and induces migration and tumor growth. J. Thorac. Oncol.

[41] Ozgur E., Mert U., Isin M., Okutan M., Dalay N., Gezer U. (2012). Differential expression of long non-coding RNAs during genotoxic stress-induced apoptosis in HeLa and MCF-7 cells. Clin. Exp. Med..

[42] Nakagawa S., Ip J.Y., Shioi G., Tripathi V., Zong X., Hirose T., Prasanth K.V. (2012). Malat1 is not an essential component of nuclear speckles in mice. RNA.

[43] Cooper C., Guo J., Yan Y., Chooniedass-Kothari S., Hube F., Hamedani M.K., Murphy L.C., Myal Y., Leygue E. (2009). Increasing the relative expression of endogenous non-coding Steroid Receptor RNA Activator (SRA) in human breast cancer cells using modified oligonucleotides. Nucleic Acids Res.

[44] Foulds C.E., Tsimelzon A., Long W., Le A., Tsai S.Y., Tsai M.J., O’Malley B.W. (2010). Research resource: Expression profiling reveals unexpected targets and functions of the human steroid receptor RNA activator (SRA) gene. Mol. Endocrinol.

[45] Amaral P.P., Neyt C., Wilkins S.J., Askarian-Amiri M.E., Sunkin S.M., Perkins A.C., Mattick J.S. (2009). Complex architecture and regulated expression of the Sox2ot locus during vertebrate development. RNA.

[46] Ishii N., Ozaki K., Sato H., Mizuno H., Saito S., Takahashi A., Miyamoto Y., Ikegawa S., Kamatani N., Hori M. (2006). Identification of a novel non-coding RNA, MIAT, that confers risk of myocardial infarction. J. Hum. Genet.

[47] Lu Y., Zhang K., Li C., Yao Y., Tao D., Liu Y., Zhang S., Ma Y. (2012). Piwil2 suppresses p53 by inducing phosphorylation of signal transducer and activator of transcription 3 in tumor cells. PLoS One.

[48] Livak K.J., Schmittgen T.D. (2001). Analysis of relative gene expression data using real-time quantitative PCR and the 2(−Delta Delta C(T)) Method. Methods.

